# Medical Commission on Tuberculosis for the State of Georgia

**Published:** 1904-10

**Authors:** 


### MEDICAL COMMISSION ON TUBERCULOSIS FOR THE STATE OF GEORGIA.

IN accordance with the provisions of a resolution passed by the last General Assembly, approved August 12, 1904, the following named persons were appointed a Commission on Tuberculosis:

From the State at large: Dr. Chas. Hicks, Dublin, Chairman; Dr. Louis H. Jones, Atlanta; Dr. Erwin P. Ham, Gainesville; Dr. P. T. Hillsman, Albany; Dr. J. R. Burdette, Tennille; Dr. Pryor H. Fitts, Greenville; Dr. S. A. Brown, Dalton; Dr. L. G. Hardman, Commerce; Dr. T. J. Belt, Millen; Dr. J. L. Walker, Waycross.

From Congressional Districts:

First—Dr. Eugene R. Corson, Savannah.

Second—Dr. O. B. Bush, Camilla.

Third—Dr. Alex Mack, Hawkinsville.

Fourth—Dr. H. R. Slack, LaGrange.

Fifth—Dr. Bernard Wolff, Atlanta.

Sixth—Dr. Geo. T. Alexander, Forsyth.

Seventh—Dr. Thos. R. Garlington, Rome.

Eighth—Dr. Henry E. Thornton, Hartwell.

Ninth—Dr. Jefferson D. Davis, Toccoa.

Tenth—Dr. Thos. D. Coleman, Augusta.

Eleventh—Dr. S. E. Sanchez, Barwick.

---

DR. L. AMSTER, after a thorough course under the best known men in Germany in the province of gastro-intestinal diseases, has returned to Atlanta and will hereafter limit his practice to this branch. He has leased and thoroughly equipped the handsome Swift residence at 267 Capitol Avenue, and will conduct a

Sanitarium for the special treatment of disorders of the stomach and intestines. The Sanitarium is beautifully situated on one of the best residence streets of Atlanta and surrounded by spacious grounds. Dr. Amster's reputation as a thorough, pains-taking, erudite and accomplished practitioner will foreshadow his success in the specialty which he has adopted, and the sole regret attached to his withdrawal from general practice will be the deprivation of his large clientele of his services in the more liberal field of miscellaneous work.

---

According to an exchange, the price of goldenseal root has been advanced nearly 100 per cent. thus far this year. The average price during January was seventy-five cents a pound, and it is now held at $1.35. The advance has been due to a scarcity of supplies, occasioned by the fact that the gathering of the root has been lighter and the consumption heavier. This is one of many indigenous drugs the shortage of which has occasioned the manufacturers of chemicals and wholesale drug dealers much inconvenience during the last year. Western senega root is another of these drugs which has been steadily advancing because of the scarcity of the supplies, and there are numerous others of lesser importance which cannot be obtained with any degree of certainty, and which are rapidly enhancing in value by reason of their scarcity.

---

There is, according to an exchange, in Paris a new school of psychology to investigate such questions as mind reading, mental suggestion at a distance, clairvoyance, presentiments, automatic writing, double personality, etc. This school, unlike most associations studying these subjects, has for its members medical men almost entirely, whose professional duties bring them in close contact with these often curious phenomena. The members are followers of Charcot.

